# Synergetic Effects of Alternating Oxidant and Carbon Supply to Enhance Defect Healing of Single‐Walled Carbon Nanotubes

**DOI:** 10.1002/smtd.202502417

**Published:** 2026-05-17

**Authors:** Man Shen, Taiki Inoue, Mengyue Wang, Yuanjia Liu, Yoshihiro Kobayashi

**Affiliations:** ^1^ Department of Applied Physics The University of Osaka Suita Osaka Japan

**Keywords:** carbon nanotube, crystallinity improvement, defect healing, diameter preservation, thermal treatment

## Abstract

The technological potential of carbon nanotubes (CNTs) is strongly limited by structural defects, which degrade their outstanding properties. Conventional strategies for post‐growth defect healing exhibit moderate efficiency and result in unfavorable structural alterations. Herein, we propose a two‐step ${\mathrm{CO}}_{2}$‐assisted multiple‐cycle strategy for the efficient and scalable healing of CNT defects while maintaining their structure. ${\mathrm{CO}}_{2}$ functions as an oxidizing agent with preferential reactivity toward defective sites such as adatoms and Stone‐Wales defects, enabling efficient etching while minimizing structural damage. In combination with $C_{2}H_{2}$, this method can substantially improve the crystallinity of CNTs, without inducing tube coalescence. The proposed approach was systematically applied to three types of single‐walled CNTs: lab‐grown CNTs, commercial eDIPS 2.0, and super‐growth CNTs. Raman spectroscopy, thermogravimetric analysis, and transmission electron microscopy confirmed the enhancement in crystallinity and thermal stability of CNTs without diameter changes, whereas isotope labeling with 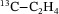 verified the improvements originated from defect healing rather than the growth of new CNTs. Notably, the proposed healing process improved the $I_{G}/I_{D}$ ratio of eDIPS 2.0 to 141, underscoring the effectiveness of this strategy. Overall, the proposed ${\mathrm{CO}}_{2}$‐assisted method shows high reproducibility, broad applicability, and superior structural preservation, establishing a scalable pathway for the post‐synthesis healing of CNTs.

## Graphical abstract

A ${\mathrm{CO}}_{2}$‐assisted multiple‐cycle healing strategy enables efficient and scalable defect healing in carbon nanotubes while preserving their intrinsic structure. By combining mild ${\mathrm{CO}}_{2}$ oxidation with $C_{2}H_{2}$, this approach achieves high crystallinity without tube coalescence, offering a generalizable post‐synthesis route toward structurally intact, high‐quality carbon nanotube materials

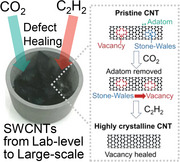

## 1 Introduction

Carbon nanotubes (CNTs), particularly single‐walled carbon nanotubes (SWCNTs) [1, 2, 3], have been extensively studied because of their exceptional mechanical strength [4, 5], excellent electrical conductivity [6], and superior thermal conductivity [7, 8]. These properties make CNTs highly promising materials for advanced technological applications, including nanoelectronics [9, 10, 11, 12, 13], sensors [14, 15], reinforced composite materials [16, 17], energy storage systems [18, 19, 20, 21], and innovative concepts such as space elevators [22, 23, 24]. However, the performance of CNTs is considerably affected by their structural integrity, as even minor defects can markedly impair their mechanical [25, 26], electrical [27, 28, 29], and thermal properties [30]. Specifically, adatom defects, which are extraneous atoms covalently bound to the CNT surface [31], substantially alter the bandgap and electronic properties of CNTs [29, 32]. Vacancy defects, including monovacancies and divacancies, arise from missing carbon atoms and severely compromise electrical performance [28, 33]. Additionally, Stone‐Wales (SW) defects, resulting from bond rotations, introduce strain into the CNT lattice [34], thereby negatively affecting mechanical resilience [35, 36]. Despite extensive research efforts dedicated to synthesizing low‐defect CNTs [37, 38, 39, 40, 41], defects are inevitably formed during the growth [42], purification [43], and dispersion processes [44]. Consequently, the development of efficient post‐synthesis defect healing methods is critical to fully exploit the technological potential of CNTs.

Thermal treatment is a commonly used approach for the healing of defects in various materials, including CNTs. For example, multi‐walled carbon nanotubes heat‐treated at 1200
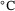
–2000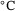 in high vacuum or at 2000
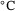
–2800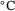 in an Ar atmosphere show enhanced crystallinity [45, 46, 47]. Similarly, high‐temperature treatment under vacuum or inert conditions has been shown to improve the crystallinity of SWCNTs; [48, 49] however, such processes typically require extreme temperatures exceeding 1800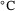. At these temperatures, the unwanted coalescence of individual CNTs often occurs, leading to considerable structural modifications [50, 51, 52]. Such structural changes are particularly detrimental for applications involving sub‐nanometer SWCNTs, where electronic properties are highly sensitive to diameter, as well as for chirality‐sorted CNTs, where maintaining the original structure is essential to preserve chirality‐dependent properties. Consequently, these structural alterations can hinder practical applications, highlighting the importance of defect healing strategies that preserve the original nanotube diameter and structure. Additionally, high‐temperature annealing can trigger carbothermal reactions in CNTs grown on oxide substrates, causing the degradation or decomposition of CNTs [53, 54]. Consequently, lower‐temperature (below 1200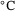) defect‐healing techniques suitable for practical use must be developed. Density functional theory simulations suggest that carbon‐based precursors, such as C_2_H_2_ and C_2_H_4_, can effectively heal vacancy defects in CNT structures [55]. Previous experimental studies have also demonstrated that C_2_H_2_ can enhance the crystallinity of CNTs at temperatures as low as 1100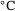 [41], whereas a multiple‐cycle defect healing involving air exposure is effective even at 1000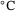 [56]. However, the healing mechanism under air exposure remains unclear, and the practical control of the process has been explored to a limited extent. Moreover, theoretical analyses indicate that C_2_H_2_ primarily heals vacancy defects, highlighting the need for strategies that can heal not only vacancies but also adatoms and SW defects [55].

**Figure 1:**
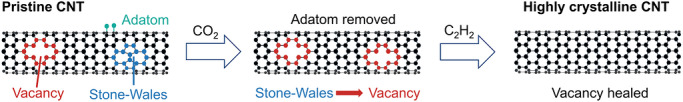
Schematic model of ${\mathrm{CO}}_{2}$‐assisted defect healing in CNTs.

Building on these findings, the present study explores the use of CO_2_ as an alternative oxidizing agent instead of air in multiple‐cycle defect healing. A new method, CO_2_‐assisted multiple‐cycle defect healing, is proposed herein, which takes advantage of the etching effect and preferential interaction of CO_2_ with defective carbon sites, such as adatoms and SW defects [57, 58]. In combination with C_2_H_2_, this approach can more efficiently heal defects at relatively low temperatures. Figure 1 shows the proposed model for ${\mathrm{CO}}_{2}$‐assisted defect healing in CNTs. Three representative defect types were considered: adatoms, vacancies, and SW defects which were employed as illustrative models for the mechanism and not individually resolved in our measurements. In the initial step of our method, ${\mathrm{CO}}_{2}$ is employed to selectively remove adatoms and convert SW defects into vacancies, as suggested by previous studies and theoretical calculations [58, 59, 60, 61]. Subsequently, $C_{2}H_{2}$ serves as a carbon source to refill the remaining vacancies, ultimately yielding highly crystalline CNTs. We comprehensively assessed the performance of the proposed strategy on three representative types of CNTs with distinct diameter distributions: lab‐grown nanodiamond CNTs (ND‐CNTs) [41, 62, 63, 64], commercially available CNTs grown through enhanced direct injection pyrolytic synthesis method (eDIPS 2.0) [65], and super‐growth CNTs (SG‐CNTs) [66, 67]. ND‐CNTs, synthesized without the use of metal catalysts, are essentially free of metallic impurities but generally possess lower crystallinity and higher defect density. eDIPS 2.0, grown via floating‐catalyst chemical vapor deposition (CVD) exhibit excellent structural quality and fewer defects but contain residual metal impurities. SG‐CNTs, produced through the “water‐assisted super‐growth” method, exhibit high aspect ratios and relatively low levels of metal impurities. These three CNT samples consist predominantly of SWCNTs. While ND‐CNTs are particularly suited for mechanistic studies on defect healing owing to their high defect concentration and metal‐free nature, eDIPS 2.0 and SG‐CNTs are high‐quality, commercially scalable CNTs with distinct growth histories and morphologies, making them promising platforms for evaluating healing performance. Since powder X‐Ray diffraction [68, 69, 70] and infrared spectroscopy [70, 71, 72] are not considered reliable methods for evaluating changes in crystallinity or defect density in SWCNTs, the healing effect was examined using Raman spectroscopy and thermogravimetric analysis (TGA), providing insights into changes in crystallinity and thermal stability. During defect healing with carbon sources, residual catalysts or growth seeds in the CNT samples may trigger the formation of new nanotubes. Consequently, high‐quality CNTs observed after healing might originate from the growth of new nanotubes rather than from the healing of pre‐existing ones. To rule out this possibility, isotope labeling with 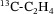 was employed. The absence of new Raman spectral features from 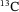
‐CNT confirmed that the proposed process does not generate new CNTs. Additional structural characterization using the radial breathing mode (RBM) analysis and transmission electron microscopy (TEM) confirmed that the nanotube diameters remain unchanged throughout the treatment. As for TEM, the combination of heaby bundling, sample thickness, and beam sensitivity of CNTs makes atomic‐scale imaging (high‐resolution TEM) extremely difficult and challenging without inducing beam damage or structural alteration. Overall, the results indicate that ${\mathrm{CO}}_{2}$‐assisted multiple‐cycle healing is effective for restoring the structure of mass‐produced CNTs as well as laboratory samples. The process enhances the crystallinity of CNTs without compromising their dimensional integrity and offers a manufacturing‐compatible, large‐batch post‐treatment pathway that may benefit a wide range of CNT‐based technologies.

## 2 Results and Discussion

### 2.1 Effect of ${\mathrm{CO}}_{2}$ Concentration on the Efficiency of Defect Healing in ND‐CNTs

**Figure 2:**
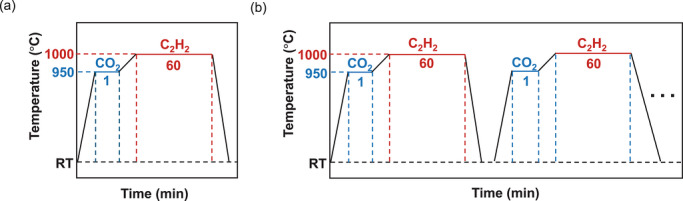
Temperature‐time schematic of (a) CO_2_‐assisted defect healing and(b) CO_2_‐assisted multiple‐cycle defect healing.

The effects of ${\mathrm{CO}}_{2}$ concentration on defect healing in ND‐CNTs were investigated. To isolate the concentration dependence from cumulative effects inherent to repeated treatments, we first assessed a single‐cycle process and subsequently extended the evaluation to multi‐cycle protocols. The temperature–time profile for the single‐cycle process is shown in Figure 2a, and the corresponding profile for the multi‐cycle process is shown in Figure 2b, with the experimental details given in the Experimental Section.

**Figure 3:**
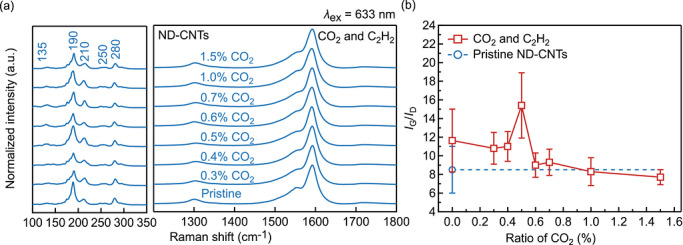
(a) Raman spectra of ND‐CNTs before and after ${\mathrm{CO}}_{2}$‐assisted defect healing with $C_{2}H_{2}$ at different ${\mathrm{CO}}_{2}$ ratios. (b) *I*_G_/*I*_D_ ratios of ND‐CNTs after ${\mathrm{CO}}_{2}$‐assisted defect healing as a function of ${\mathrm{CO}}_{2}$ concentration. The value for pristine ND‐CNTs is also shown.

Figure 3a presents the Raman spectra of ND‐CNTs before and after a single‐cycle ${\mathrm{CO}}_{2}$‐assisted defect‐healing treatment performed at different ${\mathrm{CO}}_{2}$ concentrations. The healing efficiency was evaluated using the peak intensity ratio of G‐band to D‐band, $I_{G}/I_{D}$, a widely used indicator of CNT crystallinity and defect density [73, 74, 75, 76]. The dependence of the $I_{G}/I_{D}$ ratio on ${\mathrm{CO}}_{2}$ concentration is presented in Figure 3b. Each data point was obtained from at least 10 randomly selected Raman measurements, with error bars representing the standard deviation of the $I_{G}/I_{D}$ values across different datasets. Blue circles indicate the $I_{G}/I_{D}$ values prior to treatment, whereas red squares denote the values after ${\mathrm{CO}}_{2}$‐assisted healing, highlighting the dependence of healing efficiency on ${\mathrm{CO}}_{2}$ concentration. The condition without ${\mathrm{CO}}_{2}$ is shown as 0.0% in Figure 3(b), and the effects of $C_{2}H_{2}$ alone has been reported previously [56].

As shown in Figure 3b, the $I_{G}/I_{D}$ ratio reaches its maximum at a ${\mathrm{CO}}_{2}$ concentration of 0.5%, increasing from the initial value of 8.5 to 15.4, which is higher than that obtained under $C_{2}H_{2}$ treatment alone (11.6). This indicates that a moderate ${\mathrm{CO}}_{2}$ concentration markedly enhances the healing efficiency, implying that ${\mathrm{CO}}_{2}$ functions as a mild oxidizing agent in this process. At concentrations below 0.5%, the etching effect is insufficient, leading to only marginal improvements. In contrast, concentrations above 0.5% cause excessive etching, damaging the CNT structure and reducing the healing effect. Therefore, 0.5% ${\mathrm{CO}}_{2}$ was considered the optimal concentration for efficient ${\mathrm{CO}}_{2}$‐assisted defect healing of CNTs.

The $I_{G}/I_{D}$ ratio obtained from 0.5% ${\mathrm{CO}}_{2}$ treatment alone (Supporting Information) is lower than that achieved by the combined ${\mathrm{CO}}_{2}$ and $C_{2}H_{2}$ treatment, indicating a clear cooperative effect between oxidation and carbon supply. Control experiments using blank substrates (without ND deposition) subjected to identical growth conditions show no detectable Raman signal of amorphous carbon (a‐C) (Supporting Information), suggesting that any a‐C in the pristine samples is below the detection limit. Therefore, the observed increase in $I_{G}/I_{D}$ is mainly attributed to intrinsic structural changes in the CNTs rather than the removal of external a‐C.

### 2.2 Multiple‐Cycle of ${\mathrm{CO}}_{2}$‐Assisted Defect Healing

Previous studies have shown that the healing of defects in CNTs using $C_{2}H_{2}$ exhibits a multiple‐cycle effect, where repeated treatments further increase the $I_{G}/I_{D}$ ratio beyond that achieved after a single cycle [56]. A similar trend is observed for ${\mathrm{CO}}_{2}$‐assisted defect healing. The ${\mathrm{CO}}_{2}$‐assisted multiple‐cycle defect healing process refers to the repeated application of the ${\mathrm{CO}}_{2}$‐assisted defect healing treatment. The corresponding temperature‐time schematic is shown in Figure 2b. As 0.5% ${\mathrm{CO}}_{2}$ was found to provide the best healing effect in Section 2.1, all subsequent multiple‐cycle experiments were conducted using this concentration.

**Figure 4:**
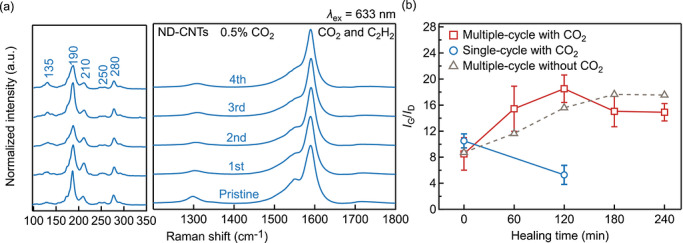
(a) Raman spectra of ND‐CNTs before and after each cycle of ${\mathrm{CO}}_{2}$‐assisted multiple‐cycle defect healing. (b) The *I*_G_/*I*_D_ ratio against healing time in multiple‐ and single‐cycle defect healing of ND‐CNTs with and without ${\mathrm{CO}}_{2}$.

Figure 4a exhibits the Raman spectra of ND‐CNTs after each cycle of ${\mathrm{CO}}_{2}$‐assisted multiple‐cycle defect healing. To assess the healing progress, the $I_{G}/I_{D}$ ratio was calculated after each cycle. The evolution of this ratio is shown in Figure 4b. The red squares show the $I_{G}/I_{D}$ values for the ${\mathrm{CO}}_{2}$‐assisted multiple‐cycle healing process. The $I_{G}/I_{D}$ increases from 8.5 to 15.4 in the first cycle and further to 18.5 in the second cycle, confirming that ${\mathrm{CO}}_{2}$‐assisted defect healing also benefits from a multiple‐cycle effect.

For comparison, the $I_{G}/I_{D}$ values after each healing cycle using only $C_{2}H_{2}$ are plotted in Figure 4b as gray triangles. In this case, three cycles were required to reach an $I_{G}/I_{D}$ of 17.6 [56], whereas the ${\mathrm{CO}}_{2}$‐assisted approach achieved a comparable value of 18.5 in just two cycles. These results indicate that the introduction of ${\mathrm{CO}}_{2}$ improves the efficiency of the defect healing process, enabling similar crystallinity to be reached with fewer treatment cycles and reduced overall processing time. This improvement in efficiency highlights the practical advantage of the ${\mathrm{CO}}_{2}$‐assisted method for accelerating defect healing. This behavior is attributed to the etching effect of ${\mathrm{CO}}_{2}$, which facilitates the removal of defective carbon sites and enhances the effectiveness of subsequent healing.

As an additional control, single‐cycle experiments with longer duration were conducted. As shown by the blue circles in Figure 4b, a single‐cycle treatment with 0.5% ${\mathrm{CO}}_{2}$ (2 min) followed by 120 min of $C_{2}H_{2}$ exposure—equivalent to the total duration of two cycles—does not enhance the $I_{G}/I_{D}$ ratio beyond the value attained through single‐cycle treatment; instead, it reduces it. Prolonged single‐cycle healing with $C_{2}H_{2}$ has been reported to cause a‐C accumulation [77], leading to reduced $I_{G}/I_{D}$ [56]. Consistent with this trend, the present single‐cycle experiments show a similar results. At the same time, the multiple‐cycle strategy suppresses a‐C deposition since it avoids long time treatment during single‐cycle, thereby enhancing the healing efficiency. These findings demonstrate that the ${\mathrm{CO}}_{2}$‐assisted multiple‐cycle strategy is a more efficient and time‐saving route for defect healing in CNTs compared to conventional $C_{2}H_{2}$ treatments.

### 2.3 Diameter Distribution Evaluated Through Raman Spectroscopy and TEM

Recently, Takakura et al. reported that in the presence of $O_{2}$, CNTs can undergo coalescence at relatively low temperatures around 600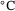, resulting in an increase in nanotube diameter [52]. As ${\mathrm{CO}}_{2}$, similar to $O_{2}$, acts as an oxidizing agent with etching capabilities, ${\mathrm{CO}}_{2}$‐treatment at temperatures below 1000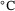 could similarly lead to an increase in CNT diameters. Therefore, we analyzed the diameter evolution during healing. Herein, both Raman spectroscopy and TEM were employed to assess the diameters of ND‐CNTs before and after ${\mathrm{CO}}_{2}$‐assisted defect healing.

It is important to note that the RBM peaks observed in Raman spectra arise from resonance effects [78]. Only CNTs with transition energies matching the laser excitation energy can exhibit RBM signals. In the present work, a laser wavelength of 633 nm (corresponding to the energy of 1.96 eV) was used. Hence, the Raman analysis was limited to CNTs that satisfy this resonance condition.

As shown in Figure 4(a), the Raman spectra of ND‐CNTs feature five distinct RBM peaks at approximately 135, 190, 210, 250, and 280 ${\mathrm{cm}}^{-1}$, corresponding to tube diameters of about 1.8, 1.2, 1.1, 0.9, and 0.8 nm (calculated using the RBM–diameter relation given in the Experimental Section). Although the RBM intensities show slight changes after defect healing, the peak positions remain nearly unchanged, indicating that the CNT diameters did not change throughout the process. Because the pre‐ and post‐treatment spectra were not acquired at the same location, each spectrum should be interpreted as an areal average over the Raman excitation spot (∼0.9 μm in diameter; $A\approx 0.81\;\mu m^{2}$). Accordingly, RBM intensity variations can also reflect fluctuations in the number of CNTs within the probed area (local tube density), whereas the near‐constant RBM frequencies indicate that the diameter distribution is essentially unchanged.

**Figure 5:**
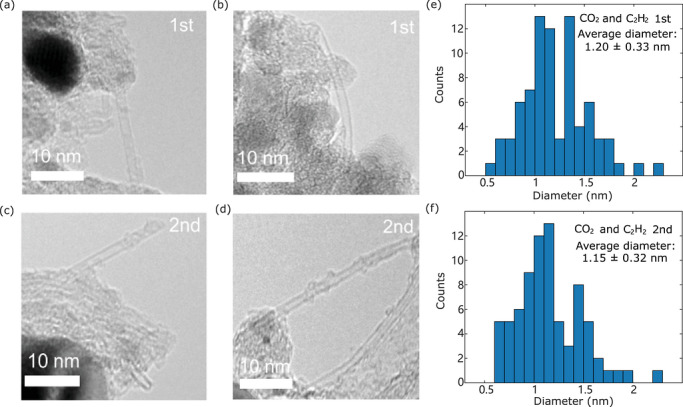
Representative TEM images of ND‐CNTs after the (a,b) 1st and (c,d) 2nd cycle of ${\mathrm{CO}}_{2}$‐assisted defect healing. Diameter distributions obtained from TEM images: (e) 1st and (f) 2nd cycle of ${\mathrm{CO}}_{2}$‐assisted defect healing.

As Raman RBM peaks do not capture all CNTs, TEM was employed to directly examine diameter distributions before and after ${\mathrm{CO}}_{2}$‐assisted healing. Figure 5 shows representative TEM images of CNTs after the first (Figure 5a,b) and second (Figure 5c,d) healing cycles. More TEM images are provided in Supporting Information. For each treatment, measurements were performed on different samples instead of tracking the same CNTs. The corresponding diameter distributions are shown in Figure 5e,f. The average diameters remain nearly unchanged–1.20±0.33 nm after the first cycle and 1.15±0.32 nm after the second, with 1.18±0.42 nm being the initial value [56].

Both Raman and TEM analyses confirm that the proposed defect‐healing method does not induce diameter enlargement in ND‐CNTs, in contrast to the behavior observed under $O_{2}$‐rich conditions reported by Takakura et al. [52] This difference was attributed to the relatively low concentration of ${\mathrm{CO}}_{2}$, as well as the random chirality and diameter of CNTs, which make coalescence difficult.

### 2.4 Absence of New CNT Growth Confirmed Through Isotope Labeling

The decrease in the D‐band intensity together with the increase in the $I_{G}/I_{D}$ indicates a reduction in the defect density of the CNTs after the healing treatment. Since Raman spectroscopy reflects the average structural information within the laser‐irradiated area, the observed decrease in defect density could in principle originate from two possible factors. The first possibility is the growth of newly formed CNTs with lower defect density, which would reduce the overall defect density within the measured region. The second possibility is the healing of pre‐existing defects in the CNT structure.To confirm that the increase in the $I_{G}/I_{D}$ ratio originates from the intrinsic improvement in crystallinity of existing CNTs rather than the growth of new CNTs, we examined whether new CNTs are formed during healing. As a carbon source is introduced during defect healing, the possibility of newly grown CNTs contributing to the observed high $I_{G}/I_{D}$ ratio cannot be ruled out without verification.

**Figure 6:**
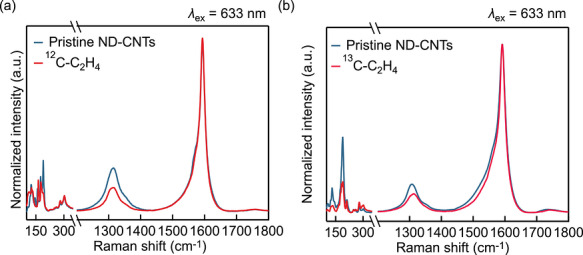
Raman spectra of ND‐CNTs before and after healing with (a) 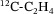 and (b) 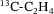.

Previous studies have shown that $C_{2}H_{2}$ and $C_{2}H_{4}$ exhibit similar defect‐healing capabilities, albeit with some differences in healing efficiency [41, 55]. Herein, we performed defect‐healing experiments using both conventional (natural‐abundance) 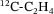 and isotopically enriched 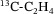. Natural‐abundance 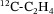 contains approximately 1 % 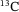, and 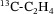 used herein has a nominal isotopic purity of 99% 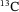. Because of the low concentration, in calculations, these samples were considered to contain 100% 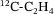 and 100% 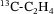, respectively. The Raman spectra before and after healing are shown in Figure 6a,b, respectively. All spectra were normalized to the G‐band intensity.

For both 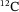
‐ and 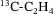, a noticeable decrease in the D‐band intensity is observed after healing, indicating an improved $I_{G}/I_{D}$ ratio and enhanced structural crystallinity of the CNTs. Importantly, in the case of 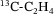, the G‐band dose not exhibit any apparent shift to lower frequency after healing. This result suggests that no new CNTs were grown during healing.

If new CNTs had been formed from the 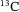 precursor, their Raman G‐band would be expected to exhibit a distinct shift to lower frequency because of the isotope effect of heavier carbon atoms [79]. According to the following equation,


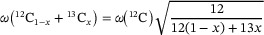

where x is the fraction of 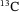 (0 ≤x≤ 1), 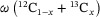 is the G‐band frequency at a given 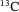 fraction, and $\omega {(}^{12}C)$ is the Raman shift for pure 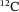
‐CNTs. The reference value of $\omega {(}^{12}C)$ is 1594 ${\mathrm{cm}}^{-1}$, as obtained from Figure 6a,b. For CNTs entirely composed of 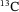 (x=1), the expected G‐band peak position, $\omega {(}^{13}C)$, is 1531 ${\mathrm{cm}}^{-1}$. The Raman spectra of CNTs grown with 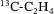, which is shown in the Supporting Information, also exhibit a G‐band peak at 1531 ${\mathrm{cm}}^{-1}$. However, no such peak shift or splitting is observed in Figure 6b, indicating that CNTs composed exclusively of 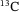 were not detected. Therefore, the observed improvement in the $I_{G}/I_{D}$ ratio was attributed solely to the enhanced crystallinity of pre‐existing CNTs rather than the formation of new CNTs.

Regarding defect healing, when vacancy defects are healed, 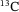 atoms from the precursor may be incorporated into the CNT structure. Nevertheless, no G‐band shift attributable to 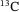 incorporation is observed, likely because of its extremely low concentration. For the pristine CNTs with $I_{G}/I_{D}=5.9$ (Figure 6b), the corresponding fractional defect density $f_{D}$ (number of defects per carbon atom) is $4.8\times 10^{-6}$. The calculation method for $f_{D}$ is provided in the Supporting Information [56, 80, 81]. After healing with 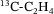, $I_{G}/I_{D}$ increased to 8.9, corresponding to $f_{D}=3.2\times 10^{-6}$. This indicates that $1.6\times 10^{-6}$ defects per carbon atom were healed. Assuming all these defects are vacancies filled by 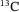, the resulting 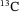 concentration would be $x=1.6\times 10^{-6}$, yielding $\omega_{\text{healed}}\approx 1594\;{\text{cm}}^{-1}$ with no detectable shift. Thus, the absence of a downshift in the G‐band can be explained by the negligible concentration of incorporated 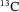. In contrast, the slight upshift of the D‐band observed after healing with 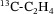 may originate from changes in the a‐C composition; however, this does not affect the conclusion that no new CNTs were formed during defect healing. Consequently, the increase in the $I_{G}/I_{D}$ ratio was attributed to the intrinsic improvement in the crystallinity of existing CNTs.

### 2.5 ${\mathrm{CO}}_{2}$‐Assisted Multiple‐Cycle Defect Healing Applied to Commercially Scalable CNTs

Defect healing of large‐scale CNT materials is essential for enabling their practical applications. Thus, we applied ${\mathrm{CO}}_{2}$‐assisted multiple‐cycle defect healing to commercially scalable CNTs (eDIPS 2.0 and SG‐CNTs), achieving effective large‐scale defect healing.

**Figure 7:**
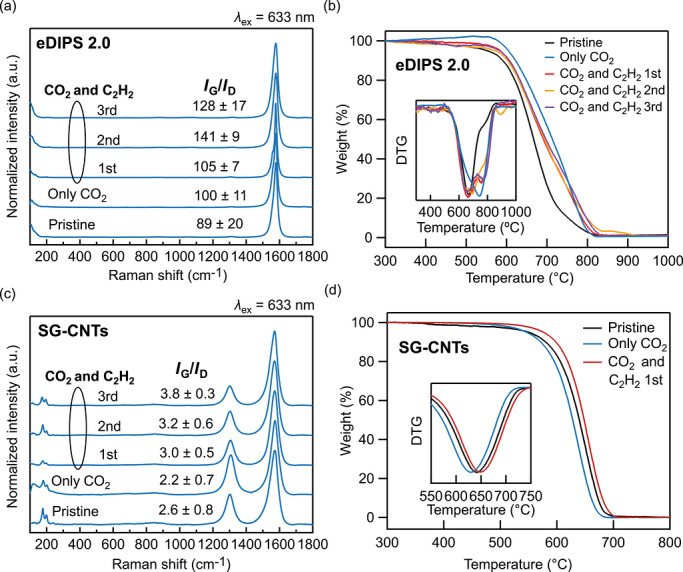
(a) Raman spectra of eDIPS 2.0 before and after the treatment with ${\mathrm{CO}}_{2}$ only and each ${\mathrm{CO}}_{2}$‐assisted healing cycle. (b) TGA and DTG (insets) curves of eDIPS 2.0 before and after the treatment with ${\mathrm{CO}}_{2}$ only and ${\mathrm{CO}}_{2}$‐assisted healing. (c) Raman spectra of SG‐CNTs before and after the treatment with ${\mathrm{CO}}_{2}$ only and each ${\mathrm{CO}}_{2}$‐assisted healing cycle. (d) TGA and DTG curves of SG‐CNTs before and after the treatment with ${\mathrm{CO}}_{2}$ only and ${\mathrm{CO}}_{2}$‐assisted healing.

As shown in Figure 7a, the Raman spectra and $I_{G}/I_{D}$ ratios of eDIPS 2.0 were analyzed before and after each ${\mathrm{CO}}_{2}$‐assisted healing cycle, whereas the data for $C_{2}H_{2}$ healing without ${\mathrm{CO}}_{2}$ and treatment with only ${\mathrm{CO}}_{2}$ are provided in Supporting Information. The initial $I_{G}/I_{D}$ ratio of eDIPS 2.0 is remarkably high (89), indicating excellent initial crystallinity. Impressively, this ratio is further enhanced to 141 after the second healing cycle. Besides, there was also a lower improvement after eDIPS 2.0 samples treated with only $C_{2}H_{2}$ or only ${\mathrm{CO}}_{2}$ compared with ${\mathrm{CO}}_{2}$ and $C_{2}H_{2}$ healing, which also indicates the combination of ${\mathrm{CO}}_{2}$ and $C_{2}H_{2}$ has better healing efficiency. Thus, our findings confirm that the proposed ${\mathrm{CO}}_{2}$‐assisted multi‐cycle process is effective in improving the crystallinity of CNTs, even those that were originally of high quality.

As the amount of ND‐CNTs obtained was too limited, TGA measurements could not be performed on these samples. In contrast, because of the relatively large quantity of eDIPS 2.0, TGA was performed as a complementary technique to Raman spectroscopy, providing an additional perspective on material quality. TGA is widely used to evaluate the thermal stability and purity of materials, thereby offering independent confirmation of improvements in CNT quality [82, 83]. As shown in Figure 7b, the TGA curves of eDIPS 2.0 shift toward higher temperatures after healing, with the corresponding differential thermogravimetry (DTG) curves exhibited in insets. This shift indicates enhanced oxidative thermal stability, which is consistent with the improvements observed through Raman analysis.

Taken together, Raman and TGA results consistently confirm that defect healing improved the crystallinity of eDIPS 2.0, validating the effectiveness of the proposed ${\mathrm{CO}}_{2}$‐assisted multiple‐cycle strategy even for commercially synthesized high‐crystallinity CNTs.

Similarly, the effect of ${\mathrm{CO}}_{2}$‐assisted healing on SG‐CNTs was investigated. As shown in Figure 7c, the $I_{G}/I_{D}$ ratio increases from 2.6 in the pristine state to 3.8 after multiple‐cycle healing. To be noticed is that, after only ${\mathrm{CO}}_{2}$ treatment, the $I_{G}/I_{D}$ ratio decreased to 2.0, which also shows the necessity of the combination of ${\mathrm{CO}}_{2}$ and $C_{2}H_{2}$. Figure 7d shows the TGA and DTG curves of SG‐CNTs before and after the treatments with ${\mathrm{CO}}_{2}$ alone and ${\mathrm{CO}}_{2}$ combined with $C_{2}H_{2}$. Treatment with ${\mathrm{CO}}_{2}$ alone shifted the curve to lower temperatures, reflecting an etching effect and reduced thermal stability. In contrast, the treatment with ${\mathrm{CO}}_{2}$ and $C_{2}H_{2}$ shifted the curve to higher temperatures, indicating the improvement in oxidative stability and crystallinity. Collectively, these results demonstrate that ${\mathrm{CO}}_{2}$‐assisted multiple‐cycle defect healing can improve the crystallinity of scalable commercial CNTs. TEM images of eDIPS 2.0 CNTs and SG‐CNTs before and after healing are shown in the Supporting Information. confirming that the initial structure of CNTs are preserved during healing process.

### 2.6 Comparison of Healing Efficiency among Three Types of CNTs

**Figure 8:**
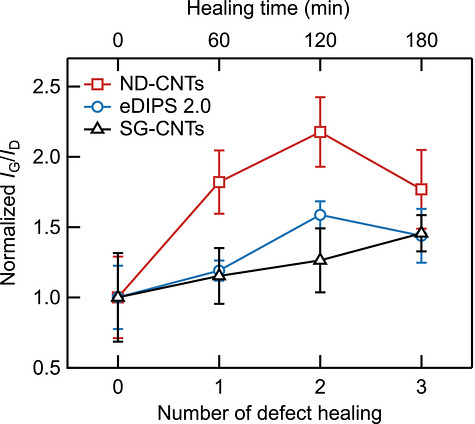
Changes in normalized *I*_G_/*I*_D_ of ND‐CNTs, eDIPS 2.0, and SG‐CNTs with increasing number of ${\mathrm{CO}}_{2}$‐assisted defect healing cycles.

We compared the effectiveness of ${\mathrm{CO}}_{2}$‐assisted multiple‐cycle defect healing on different types of CNTs. Figure 8 shows the evolution of normalized $I_{G}/I_{D}$ for three CNT types: ND‐CNTs (red squares), eDIPS 2.0 (blue circles), and SG‐CNTs (black triangles). To direct compare healing efficiency across samples with different initial defect levels, the normalized $I_{G}/I_{D}$ was obtained by dividing each value by the initial $I_{G}/I_{D}$ of respective CNTs.

The experimental results reveal a correlation between healing efficiency and CNT diameter. Among the three CNT types, ND‐CNTs, which have the smallest average diameter (1.2 nm), exhibit the most pronounced improvement in crystallinity. Their normalized $I_{G}/I_{D}$ reaches 2.2 after the second cycle, corresponding to a reduction in defect density to 45.5% of the initial level. In contrast, the larger‐diameter eDIPS 2.0 (2–3 nm) and SG‐CNTs (3–5 nm) show smaller increases in the normalized ratio, reaching 1.6 and 1.4, which correspond to defect densities reduced to 62.5% and 71.4% of the initial level, respectively. This indicates that the proposed defect healing method is more effective for CNTs with smaller diameters.

The observed differences in healing efficiency were primarily attributed to the diameter‐dependent properties of CNTs. Whereas the initial defect density can potentially affect the healing process, our use of a normalized $I_{G}/I_{D}$ ratio minimized this effect, allowing for a direct assessment of the healing efficiency. A plausible explanation for this trend is that smaller‐diameter CNTs possess larger curvature and higher local strain [84]. These characteristics are known to enhance the chemical reactivity and accessibility of defect sites to reactive species [41, 85, 86]. This enhanced reactivity explains why ND‐CNTs, being the smallest in diameter, were the most responsive to the healing treatment. Nevertheless, all samples showed an increase in $I_{G}/I_{D}$, confirming that the proposed method is effective across a wide range of CNT morphologies, including both film and bulk‐scale powders.

## 3 Conclusion

This study proposes a ${\mathrm{CO}}_{2}$‐assisted multiple‐cycle defect healing method that effectively enhances the crystallinity of various types of CNTs, including both lab‐synthesized ND‐CNTs and commercially available CNT powders such as eDIPS 2.0 and SG‐CNTs. For ND‐CNTs, 0.5% ${\mathrm{CO}}_{2}$ concentration produced a marked healing effect and shortened the treatment duration, increasing the $I_{G}/I_{D}$ ratio from 8.5 to 18.5, corresponding to a 54.5% reduction in defect density.

RBM analysis and TEM observations confirmed that the CNT diameters remained unchanged after healing, indicating that the ${\mathrm{CO}}_{2}$‐assisted multiple‐cycle treatment improves the crystallinity of CNTs without altering the nanotube diameter. Furthermore, experiments involving isotopically labeled 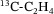 verified that no new CNTs were formed during healing. The lack of observable shift to lower frequency or peak splitting in the G‐band supports the conclusion that the enhancement in crystallinity originates from the healing of pre‐existing CNTs rather than the formation of new ones.

For eDIPS 2.0, the $I_{G}/I_{D}$ ratio reached 141 under 633 nm excitation. TGA results also confirmed the improvement in CNT quality for eDIPS 2.0 and SG‐CNTs. Among ND‐CNTs, eDIPS 2.0, and SG‐CNTs, smaller‐diameter CNTs exhibited greater healing efficiency, whereas larger‐diameter CNTs showed more modest improvement. Importantly, all three CNT types showed an increase in $I_{G}/I_{D}$ after healing, indicating the compatibility of the proposed method with large‐batch production and multiple synthesis routes.

Overall, the ${\mathrm{CO}}_{2}$‐assisted multiple‐cycle defect‐healing strategy is a promising, generalizable, and scalable post‐growth treatment that improves the structural quality of CNTs while preserving their intrinsic structure. Its robustness across different CNT sources suggests straightforward integration into manufacturing workflows and broad potential for applications in electronics, energy storage, and composite materials.

## 4 Experimental Section

### 4.1 Preparation of ND‐CNTs, eDIPS 2.0, and SG‐CNTs

ND‐CNTs were prepared following established protocols [41, 56, 62, 64]. Briefly, silicon substrates with a 300 nm thermally grown SiO_2_ layer were seeded with purified ND particles supplied by Nippon Kayaku. To remove surface contaminants, the substrates were annealed in air at 600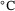 for 10 min prior to CNT synthesis. CNTs were synthesized in a three‐zone CVD furnace, with respective zone temperatures set to 850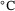, 785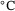, and 750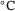. The reaction proceeded for 10 min under 10 sccm C_2_H_2_ (2% in Ar) and 10 sccm H_2_ (3% in Ar) flow. The resulting ND‐CNTs were characterized by Raman spectroscopy prior to use in the defect healing experiments.

Commercially available eDIPS 2.0 powder (EC2.0‐P), synthesized via floating catalyst CVD (purity exceeding 98%), were purchased from Meijo Nano Carbon and used without further purification. For each healing experiment, approximately 4 mg of eDIPS 2.0 was placed in a graphite crucible, which was partially covered with a graphite lid (leaving one‐quarter of the opening exposed) to reduce material loss. The crucible was then placed in a furnace and subjected to defect healing under specified conditions.

SG‐CNT powder (ZEONANO SG101), synthesized via the super‐growth method (water‐assisted CVD) [66], was obtained from Zeon Nano Technology Co., Ltd. For the healing treatment, approximately 5 mg of SG‐CNTs was loaded into a graphite crucible, which was partially covered with a graphite lid leaving one‐quarter of the opening exposed to prevent material from being blown away. The crucible was then placed in the furnace for defect healing under controlled conditions.

### 4.2 Single‐ and Multiple‐Cycle Defect Healing

The proposed multiple‐cycle defect healing treatment is based on our previously published method [56]. In the present study, we proposed and examined a newly developed CO_2_‐assisted multiple‐cycle defect healing process.

For the experimental procedure of ${\mathrm{CO}}_{2}$‐assisted single‐cycle defect healing (Figure 2(a)), CNTs were either placed on substrates or loaded into graphite crucibles, followed by heating in a CVD furnace under a 25 sccm Ar flow at 65 Pa until the temperature reached 950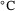. At this point, a gas mixture consisting of 0.08‐0.38 sccm ${\mathrm{CO}}_{2}$ (0.3–1.5%) and 25 sccm Ar was introduced for 1 min, corresponding to a ${\mathrm{CO}}_{2}$ partial pressure of 0.20–0.98 Pa. The temperature was then increased to 1000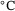 under continuous Ar flow. Upon reaching 1000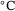, defect healing was initited through the introduction of a gas mixture containing 5 sccm $C_{2}H_{2}$ (2% in Ar) and 20 sccm Ar, maintaining the total pressure at 65 Pa. This setup resulted in a partial pressure of 0.26 Pa for $C_{2}H_{2}$. The healing step lasted for 60 min. After the process, the furnace was allowed to cool to room temperature (RT, 25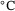) under a steady flow of 20 sccm Ar. Once cooled, the CNT samples were retrieved for characterization. In the case of ${\mathrm{CO}}_{2}$‐assisted multiple‐cycle defect healing, this sequence was repeated for subsequent healing cycles by reintroducing the CNTs into the furnace (Figure 2b).

### 4.3 Defect Healing on ND‐CNTs Using Both and Isotopically Labeled 


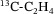
 (2%)/Ar was purchased from SUZUKI SHOKAN Co., Ltd. For the treatments with 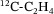 and isotopically labeled 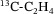, ND‐CNTs were heated in the CVD furnace under an Ar flow of 25 sccm at a pressure of 65 Pa until the temperature reached 1000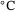. Upon reaching this temperature, the defect‐healing process was initiated through the introduction of a gas mixture consisting of 3.5 sccm $C_{2}H_{4}$ (2%)/Ar and 22 sccm Ar. The total pressure during healing process was maintained at 65 Pa, corresponding to a $C_{2}H_{4}$ partial pressure of 0.18 Pa.

### 4.4 Characterization

Raman spectra of SWCNTs before and after defect healing were acquired using a Raman spectrometer (LabRAM HR800, HORIBA Jovin Yvon) at an excitation wavelength of $\lambda_{\mathrm{ex}}=633$ nm. The laser spot size was approximately 0.9 μm, and the laser power at the measurement point was maintained at around 9 mW. Each spot was exposed for 1 s over five accumulations. For each sample, spectra were collected from at least ten randomly selected locations and averaged for subsequent analysis. The structural quality of the CNTs was evaluated using the peak intensity ratio of the G‐band (around 1590 ${\mathrm{cm}}^{-1}$) to the D‐band (1300–1350 ${\mathrm{cm}}^{-1}$), denoted as $I_{G}/I_{D}$ [73, 74, 75, 76]. The $I_{G}/I_{D}$ ratio was further used to estimate defect density based on previous studies [80, 81, 87].

The RBMs, observed between 100 and 300 ${\mathrm{cm}}^{-1}$, were analyzed to determine the SWCNT diameter ($d_{t}$) using the following equation [88]:

$$\omega_{\mathrm{RBM}}\left({\mathrm{cm}}^{-1}\right)=217.8/d_{t}\left(\mathrm{nm}\right)+15.7$$

Morphological characterization was performed using TEM (JEM‐2100 Plus, JEOL) at an acceleration voltage of 200 kV. For TEM analysis, ND‐CNTs were sonicated in ethanol for 1 h, and the resulting dispersion was drop‐cast onto a copper grid coated with a holey carbon film.

TGA was conducted using a TG8120 analyzer (Rigaku) under a continuous flow of dry air at 200 sccm. Sample masses ranged from 1.0 to 1.5 mg. The eDIPS 2.0 samples were heated to 1000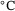 at a rate of 5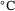/min, whereas the SG‐CNT samples were heated to 800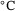 at the same rate.

## Conflicts of Interest

The authors declare no conflicts of interest.

## Supporting information


**Supporting File**: smtd70714‐sup‐0001‐SuppMat.pdf.

## Data Availability

The data that support the findings of this study are available from the corresponding author upon reasonable request.
