# Supplementary material for: Synergetic Effects of Alternating Oxidant and Carbon Supply to Enhance Defect Healing of Single‐Walled Carbon Nanotubes
Source: Small Methods. 2026 May 17;10(11):e02417. doi: 10.1002/smtd.202502417 (PMC13244295; doi:10.1002/smtd.202502417)
Supplement: Supplementary file 1 — Supporting File: smtd70714‐sup‐0001‐SuppMat.pdf. [file SMTD-10-e02417-s001.pdf]

Supporting Information

Synergetic Effects of Alternating Oxidant and Carbon Supply to Enhance Defect Healing of Single-Walled Carbon Nanotubes

Man Shen*[1], Taiki Inoue[1], Mengyue Wang[1], Yuanjia Liu[1] and Yoshihiro Kobayashi*[1]

[1]Department of Applied Physics, The University of Osaka, Suita, Osaka, 565-0871, Japan

E-mail: shen.m@ap.eng.osaka-u.ac.jp; kobayashi@ap.eng.osaka-u.ac.jp

Growth of CNTs from $^{13}C\text{-}C_2H_4$

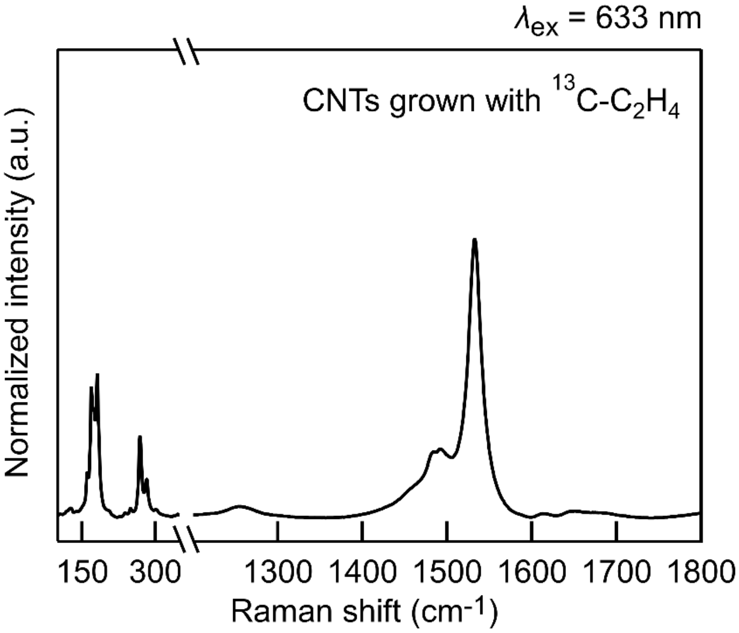

Figure S1. Raman spectra of CNTs grown from $^{13}C\text{-}C_2H_4$.

Raman spectra of eDIPS 2.0 with C_2H_2 healing

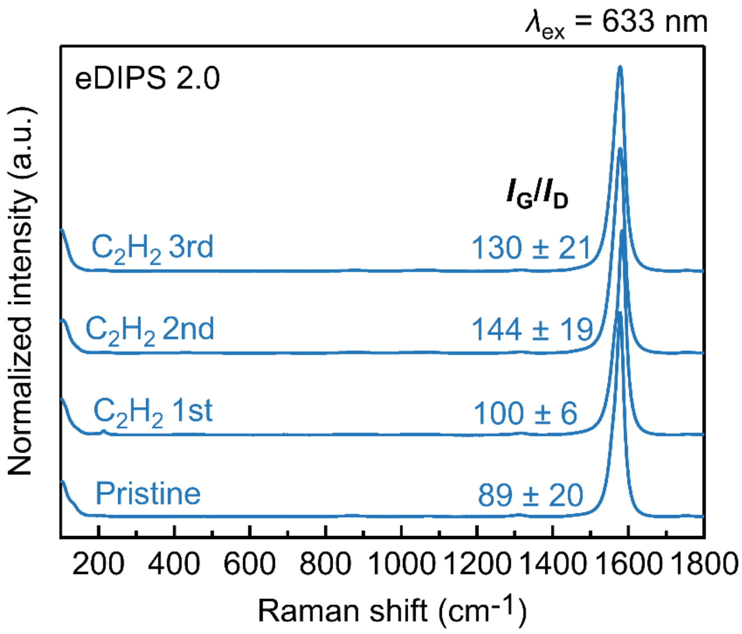

Figure S2. Raman spectra of eDIPS 2.0 before and after each healing cycle with C_2H_2 healing.

Evolution of I_G/I_D ratio of eDIPS 2.0 with and without CO_2

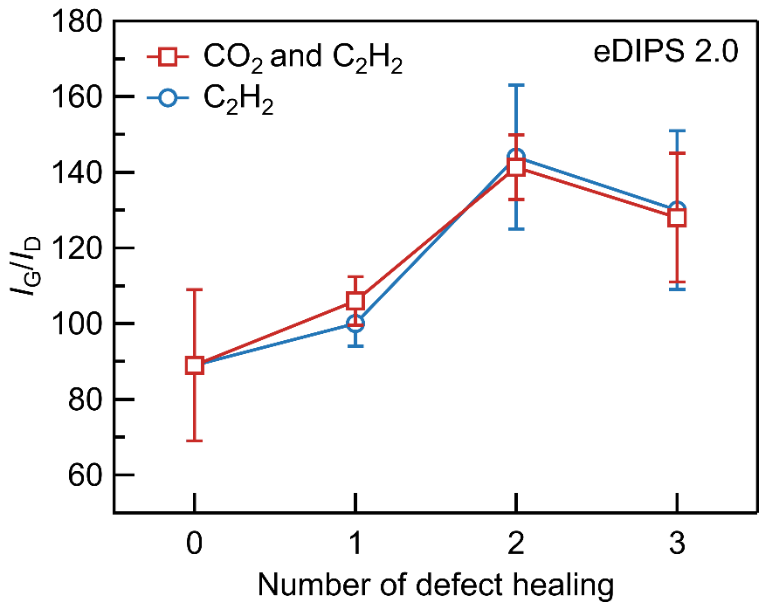

Figure S3. Evolution of I_G/I_D ratio of eDIPS 2.0 as a function of healing cycles of multiple-cycle defect healing with and without CO_2.

Although some differences exist between the two cases, the overall healing efficiency is comparable. This observation suggests that for eDIPS 2.0, the presence of CO_2 does not significantly alter the healing outcome, implying that healing performance can vary depending on CNT type, even under the same treatment conditions. It should be noted that 0.5% CO_2 was optimized for ND-CNTs and directly applied to eDIPS 2.0 and SG-CNTs, while optimization for these two CNTs remains to be explored.

TGA and DTG curves of SG-CNTs

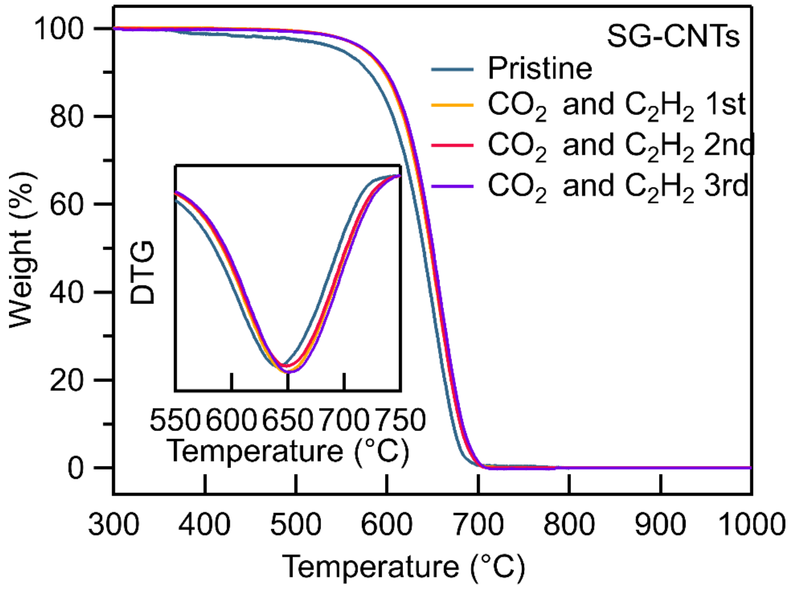

Figure S4. Thermogravimetric analysis (TGA) and derivative thermogravimetry (DTG, insets) curves of SG-CNTs before and after healing.

TEM images of eDIPS 2.0 and SG-CNTs before and after defect healing

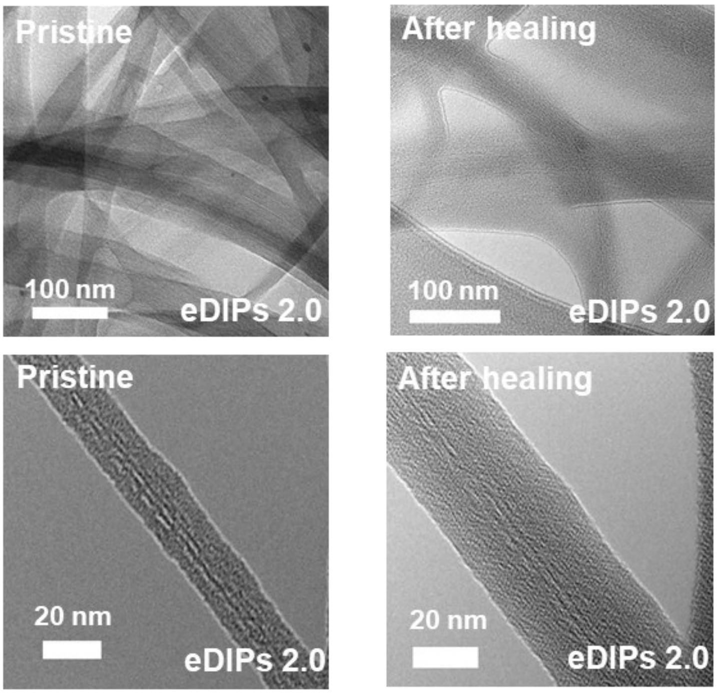

Figure S5: TEM images of eDIPS 2.0 before and after defect healing with CO_2 and C_2H_2.

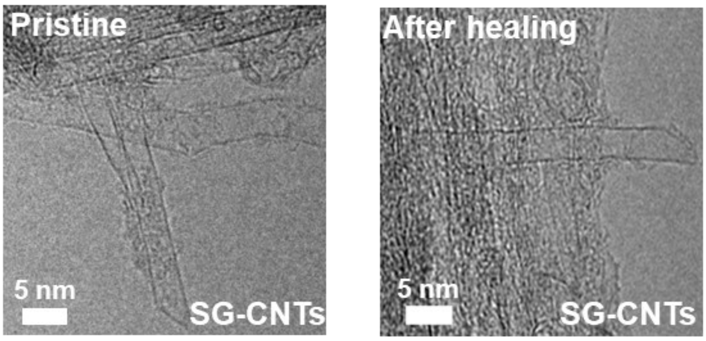

Figure S6: TEM images of SG-CNTs before and after defect healing with CO_2 and C_2H_2.

Comparison of I_G/I_D after treatment with only CO_2 and CO_2 and C_2H_2

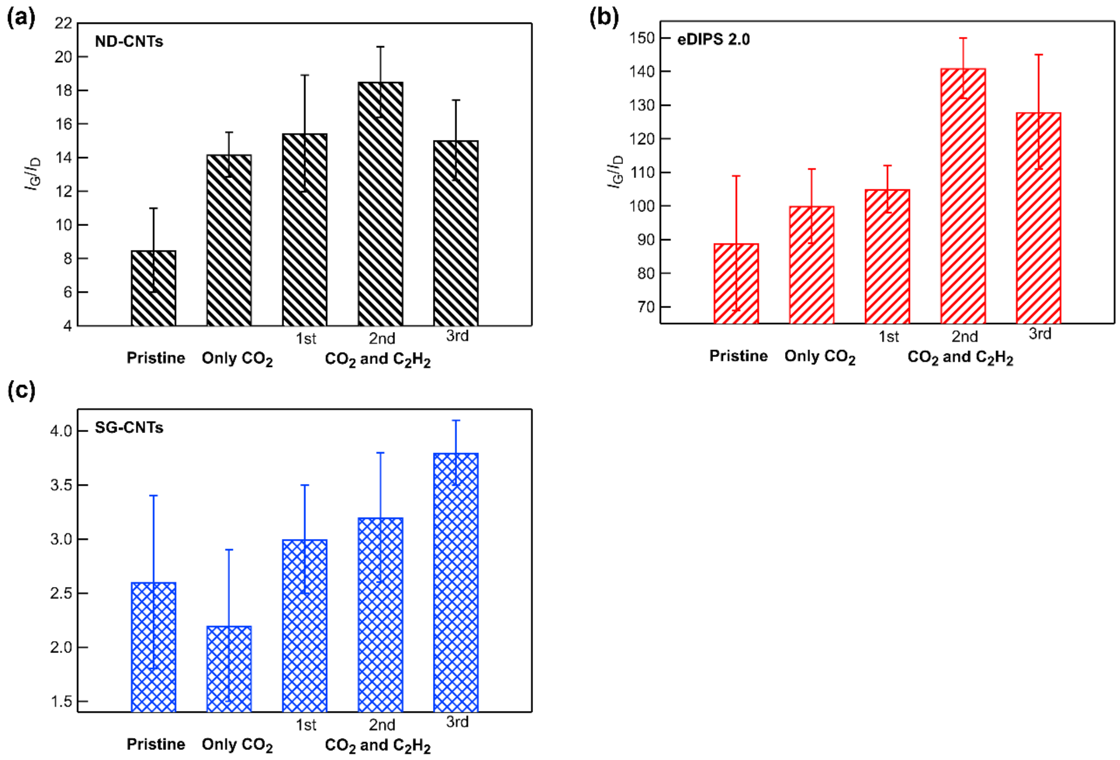

Figure S7: Change of I_G/I_D ratio of ND-CNTs before and after the treatment with CO_2 only and the first cycle of CO_2-assisted healing.

Raman spectra of blank sample

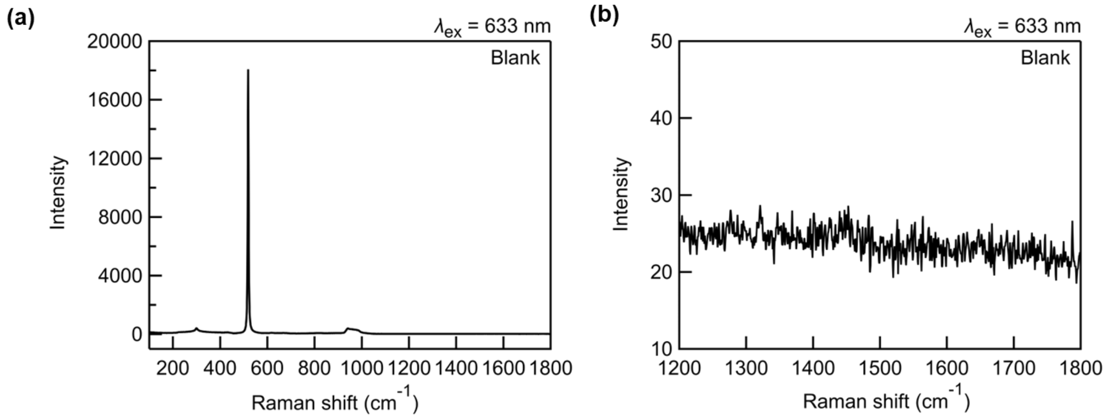

Figure S8: Raman spectra of the blank sample: (a) full spectral range (100 to 1800 cm-1) and (b) magnified view of the D- and G-band region (1200 to 1800 cm-1).

TEM images of ND-CNTs

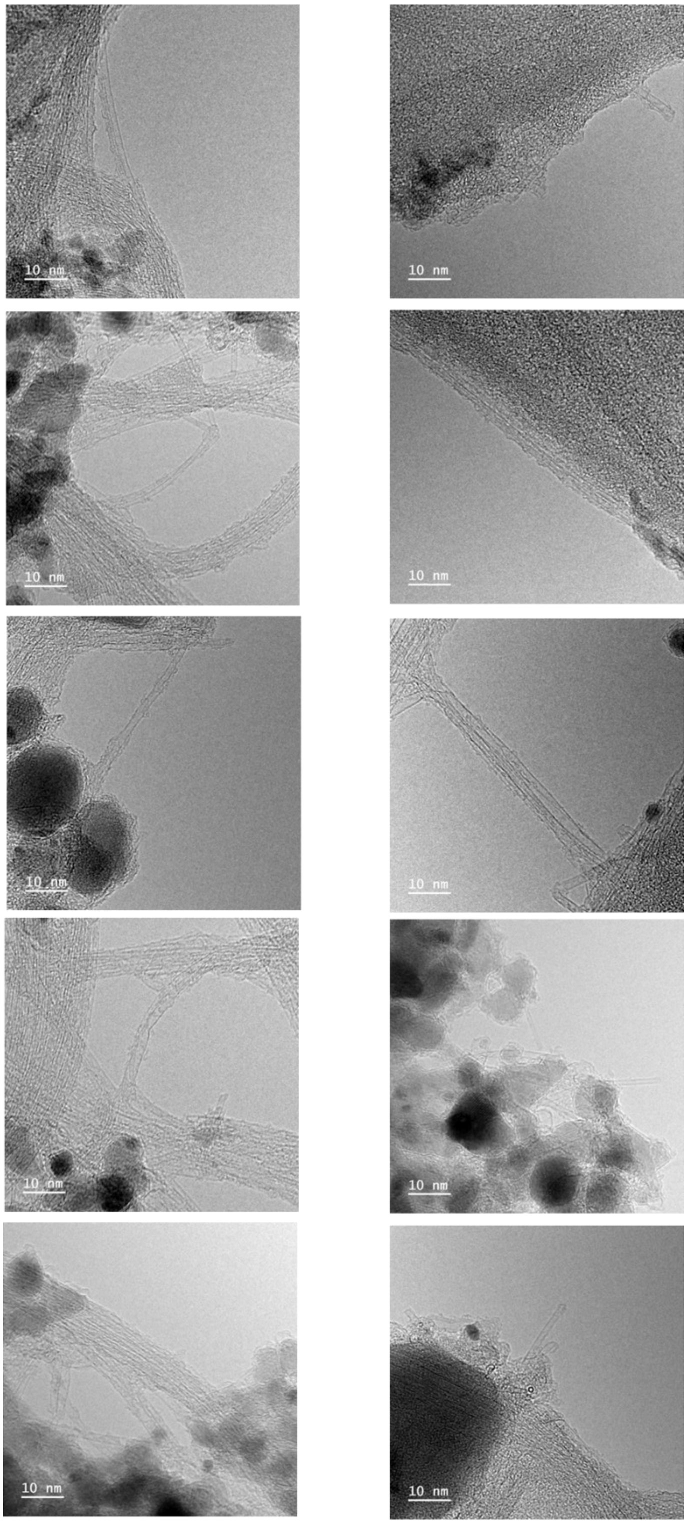

Figure S9: TEM images of pristine ND-CNTs obtained from ten different regions.

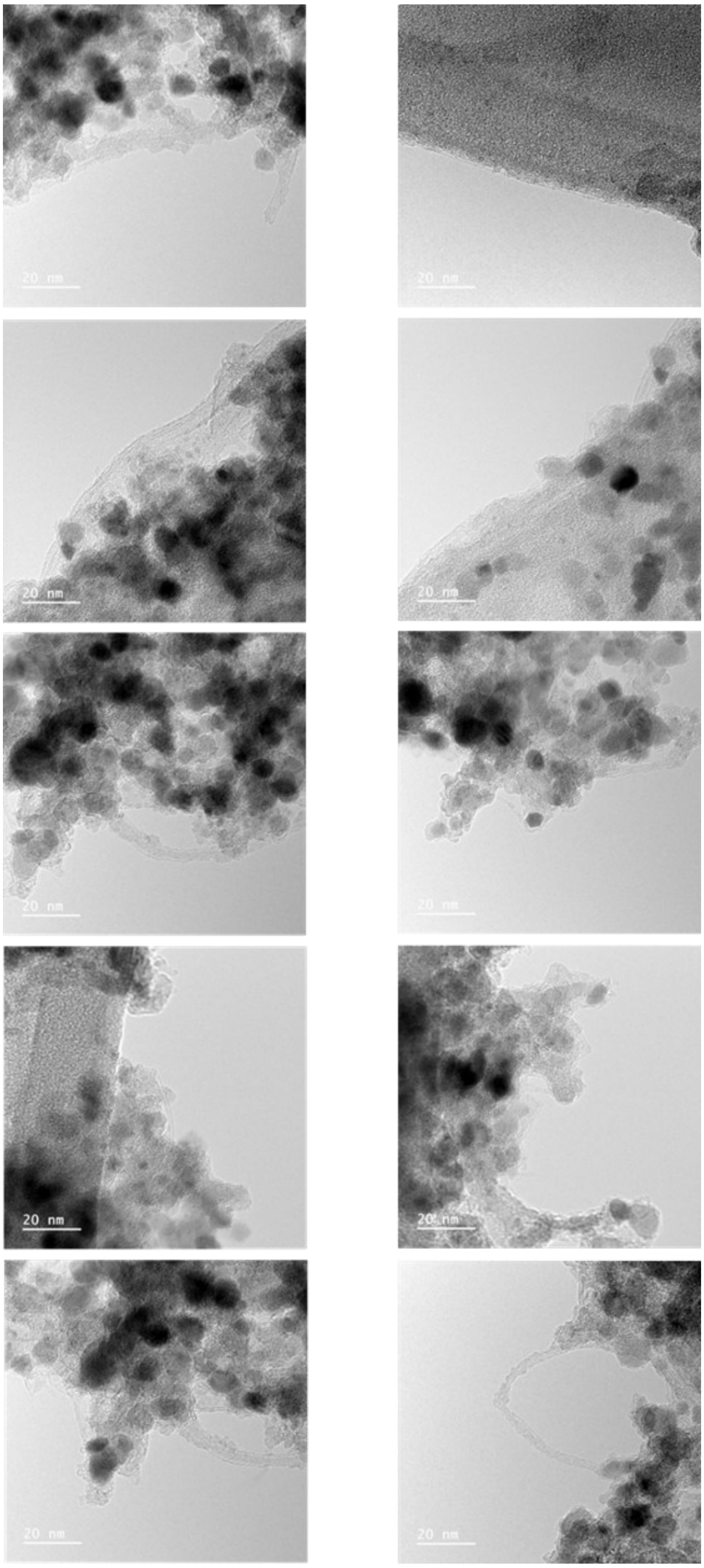

Figure S10: TEM images of ND-CNTs after the first healing cycle obtained from ten different regions.

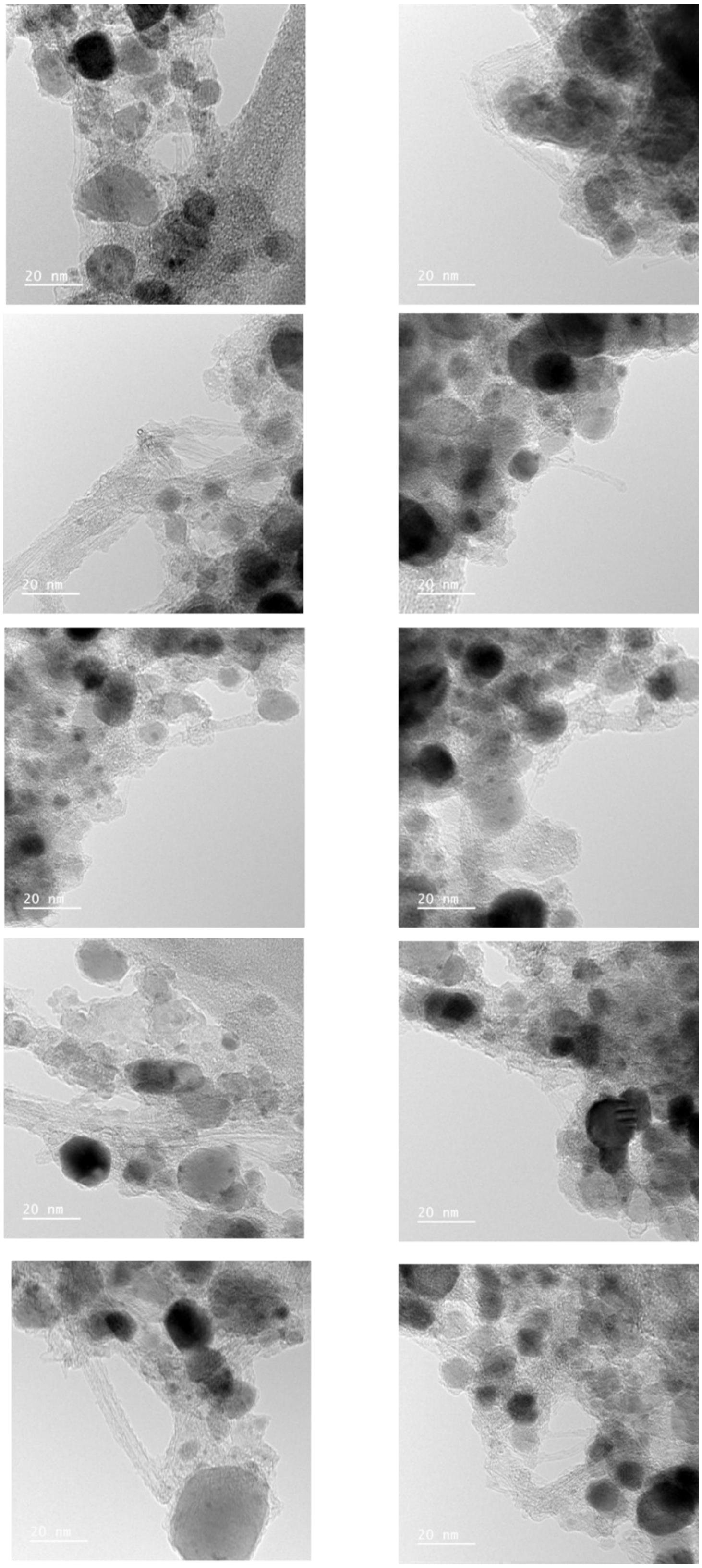

Figure S11: TEM images of ND-CNTs after the second healing cycle obtained from ten different regions.

Estimation of defect density

According to the three references, for SWCNTs, the defect distance L_D is given by:

$$L_D^2\ (\text{nm}^2) = \frac{(4.3 \pm 1.3) \times 10^3}{E_l^4}\ (I_G/I_D)$$

where E_l is the excitation laser energy used in the Raman experiment in eV units.
The defect density n_D is given by the equation:

$$n_D\ (\text{nm}^{-2}) \approx 1/\ (\pi \times L_D^2)$$

and the linear defect density λ_D (along the length of a SWCNT of diameter d nm) is given by the equation:

$$\lambda_D\ (\text{defect/nm}) = \pi d n_D$$

the fractional defect density f_D (number of defects per carbon atom) is given by the equation:

$$f_D\ (\text{defect/carbon atom}) = \Omega n_D$$

where Ω = 0.026 nm^2/carbon is the density of graphene.
For a laser with wavelength = 633 nm, E_{633} = 1.96 eV, the average diameter of ND-CNTs is 1.2 nm and we can estimate defect distance L_D, defect density n_D, linear defect density λ_D and fractional defect density f_D from the above equations using I_G/I_D.

References:

(1) Cançado, L. G., et al. "General equation for the determination of the crystallite size L_a of nanographite by Raman spectroscopy." *Applied Physics Letters* 88.16 (2006).

(2) Cançado, L. Gustavo, et al. "Quantifying defects in graphene via Raman spectroscopy at different excitation energies." *Nano letters* 11.8 (2011): 3190-3196.

(3) Vinten, Phillip, et al. "Thermodynamic and energetic effects on the diameter and defect density in single-walled carbon nanotube synthesis." *The Journal of Physical Chemistry C* 117.7 (2013): 3527-3536.
